# Patch clamp studies of human sperm under physiological ionic conditions reveal three functionally and pharmacologically distinct cation channels

**DOI:** 10.1093/molehr/gau003

**Published:** 2014-01-16

**Authors:** S.A. Mansell, S.J. Publicover, C.L.R. Barratt, S.M. Wilson

**Affiliations:** 1 Medical Research Institute, College of Medicine, Dentistry and Nursing, Ninewells Hospital and Medical School, University of Dundee, Dundee DD1 9S, UK; 2 School of Biosciences, The University of Birmingham, Birmingham B15 2TT, UK; 3 Wolfson Research Institute, School of Medicine, Pharmacy and Health, Queen's Campus, University of Durham, Stockton on Tees TS17 6BH, UK

**Keywords:** CatSper, fertilization, patch clamp recording, spermatozoa, K^+^ channel

## Abstract

Whilst fertilizing capacity depends upon a K^+^ conductance (*G*_K_) that allows the spermatozoon membrane potential (*V*_m_) to be held at a negative value, the characteristics of this conductance in human sperm are virtually unknown. We therefore studied the biophysical/pharmacological properties of the K^+^ conductance in spermatozoa from normal donors held under voltage/current clamp in the whole cell recording configuration. Our standard recording conditions were designed to maintain quasi-physiological, Na^+^, K^+^ and Cl^−^ gradients. Experiments that explored the effects of ionic substitution/ion channel blockers upon membrane current/potential showed that resting *V*_m_ was dependent upon a hyperpolarizing K^+^ current that flowed via channels that displayed only weak voltage dependence and limited (∼7-fold) K^+^ versus Na^+^ selectivity. This conductance was blocked by quinidine (0.3 mM), bupivacaine (3 mM) and clofilium (50 µM), NNC55-0396 (2 µM) and mibefradil (30 µM), but not by 4-aminopyridine (2 mM, 4-AP). Progesterone had no effect upon the hyperpolarizing K^+^ current. Repolarization after a test depolarization consistently evoked a transient inward ‘tail current’ (*I*_Tail_) that flowed via a second population of ion channels with poor (∼3-fold) K^+^ versus Na^+^ selectivity. The activity of these channels was increased by quinidine, 4-AP and progesterone. *V*_m_ in human sperm is therefore dependent upon a hyperpolarizing K^+^ current that flows via channels that most closely resemble those encoded by *Slo3*. Although 0.5 µM progesterone had no effect upon these channels, this hormone did activate the pharmacologically distinct channels that mediate *I*_Tail_. In conclusion, this study reveals three functionally and pharmacologically distinct cation channels: *Ik, I*_Tail_, *I*_CatSper_.

## Introduction

Plasma membrane ion channels are central to the control of sperm function (Darszon *et al.*, 1999; Lishko *et al.*, 2011b) and, in particular, Ca^2+^ entry via sperm cation channels (CatSper) is critical for several physiologically important processes, including hyperactivation, chemotaxis and the acrosome reaction (Lishko *et al.*, 2011a, b; Strünker *et al.*, 2011; Brenker *et al.*, 2012). Like somatic cells, mouse and human spermatozoa normally display negative resting membrane potentials (*V*_m_) that are dependent upon the activity of K^+^ channels, and the magnitude of this potential exerts a strong influence over Ca^2+^ influx since it determines the gating of CatSper and also sets the driving force for Ca^2+^ entry through these channels. At least in mouse sperm, a negative shift in *V*_m_ (hyperpolarization) is essential to capacitation, the acquisition of fertilizing ability that occurs within the female reproductive tract (Zeng *et al.*, 1995; De la Vega-Beltran *et al.*, 2012). Understanding the mechanisms that allow *V*_m_ to be maintained is therefore central to our understanding of spermatozoon physiology.

Whilst protein and mRNA encoding several K^+^ channel subtypes, including voltage-gated K^+^ channels (KCNA5) (Felix *et al.*, 2002), tandem pore domain K^+^ channels (KCNK5) (Barfield *et al.*, 2005a, b) and ATP-gated K^+^ channels (Acevedo *et al.*, 2006; Martínez-López *et al.*, 2009), is present in mouse and human sperm, the biophysical properties of K^+^ channels in these cells are only just becoming clear. Electrophysiological studies of mouse sperm thus led to the identification of the sperm K^+^ channel (KSper), a K^+^-permeable conductance whose activity was strongly enhanced by intracellular alkalinization (Navarro *et al.*, 2007). KSper-dependent K^+^ currents apparently flow via channels encoded by *Slo3* (*KCNMA3*) (Navarro *et al.*, 2007; Santi *et al.*, 2010; Zeng *et al.*, 2011), a gene expressed only in male germ cells (Schreiber *et al.*, 1998; Santi *et al.*, 2010; Yang *et al.*, 2011; Zeng *et al.*, 2011). *Slo3*-encoded channels resemble the endogenous mouse K^+^ channels in their pharmacology, weak voltage sensitivity, low K^+^ versus Na^+^ selectivity and sensitivity to changes in intracellular pH (pH_i_) (Schreiber *et al.*, 1998; Zhang *et al.*, 2006a, b; Martínez-López *et al.*, 2009). Moreover, *V*_m_ in mouse sperm is clearly dependent upon pH_i_, an observation consistent with a principal role for *Slo3* in the mature spermatozoon (Navarro *et al.*, 2007; Martínez-López *et al.*, 2009). *Slo3* gene deletion thus abolishes the hyperpolarization seen during capacitation and mimics the effects of K^+^ channel blockade on sperm function (Santi *et al.*, 2010; Zeng *et al.*, 2011). Very recent studies of human sperm, on the other hand, suggest that the K^+^ conductance of these cells is insensitive to changes in pH_i_ but enhanced by high intracellular Ca^2+^ (50 µM). These authors therefore suggested that the principal K^+^ channel in human sperm is the large conductance, Ca^2+^-sensitive (BK) K^+^ channel encoded by the *Slo1* gene (Mannowetz *et al.*, 2013). In neurons, these channels regulate excitability and control [Ca^2+^]_i_ by opening in response to increased [Ca^2+^]_i_, causing a negative shift in membrane potential which ‘switches off’ voltage-sensitive Ca^2+^ channels (Hoshi, 2012).

Whilst the present study also uses the whole cell recording technique to characterize the K^+^ channels in human sperm, our data suggest that K^+^ currents flow via a population of channels that displays relatively poor ionic selectivity, a feature that is not consistent with a central role for *Slo1* encoded channels. In addition, we identify a second, poorly selective, voltage-sensitive cation conductance whose activity is potentiated by progesterone but shows clear pharmacological difference to CatSper.

## Materials and Methods

### Experimental solutions

All concentrations are in mM. Synthetic human tubular fluid (HTF): NaCl, 97.8; KCl, 4.69; MgSO_4_; 0.2; CaCl_2_, 2.04; HEPES, 21; Glucose, 2.78; Lactic acid; 21.4; Na-pyruvate, 0.33; pH adjusted to 7.4 with NaOH. Capacitating medium: NaCl, 135; KCl, 5, MgSO_4_, CaCl_2_, 2; HEPES, 20; Glucose, 5; Lactic acid, 10; Na-Pyruvate, 1; NaHCO_3_, 25; fetal bovine serum, 20%; pH adjusted to 7.4 with NaOH. Standard bath solution: NaCl, 135, KCl, 5, CaCl_2_, 2; MgSO_4_, 1; HEPES, 20, Glucose, 5, Na pyruvate, 1; Lactic acid, 10; pH adjusted to 7.4 with NaOH which brought [Na^+^] to 154 mM. The K^+^-rich bath solution ([K^+^] = 130 mM) was prepared by iso-osmotically replacing most Na^+^ with K^+^ whilst the low Na^+^ ([Na+] = 11 mM) solution was prepared by iso-osmotically replacing Na^+^ with *N*-methyl-d-glucammonium (NMDG^+^). The divalent-free bath solution was prepared by omitting CaCl_2_ and MgCl_2_ and adding 1 mM EGTA. Standard pipette solution: NaCl, 10; KCl, 18; K gluconate, 92; MgCl_2_, 0.5, CaCl_2_, 0.6; EGTA, 1; HEPES, 10; pH adjusted to 7.4 using KOH which brought [K^+^] to 114 mM and [Ca^2+^] to 0.1 µM. For some experiments, the pH of this solution was adjusted to values between 6.2 and 8.0 and, for these experiments, pH was buffered using 5 mM MES/5 mM HEPES. Moreover, since the ability of EGTA to buffer Ca^2+^ is pH-dependent, the amount of CaCl_2_ added to these solutions was adjusted in order to maintain [Ca^2+^]_i_ at 0.1 µM irrespective of pH. K^+^-free pipette solutions were prepared by iso-osmotically replacing K^+^ with Cs^+^, Na^+^ or NMDG^+^. Non-selective cation currents flowing via spermatozoon cation channels (CatSper) were quantified using pipette (Cs-methanesulphonate, 130; HEPES, 40; Tris–HCl, 1; EGTA, 3; EDTA, 2 mM, pH adjusted to 7.4 with CsOH) and bath (Cs-methane sulphonate, 140; HEPES, 40; EGTA, 3; pH adjusted to 7.4 with CsOH) solutions devoid of Ca^2+^ and Mg^2+^ that contained Cs^+^ as the principal cation; the rationale underlying the design of these solutions is presented elsewhere (Kirichok *et al.*, 2006; Lishko *et al.*, 2011a).

### Preparation of spermatozoa

Semen samples were provided by volunteer donors with no known fertility problems after 48–72 h of sexual abstinence. All donors were shown to produce normal semen (i.e. ≥ 32% progressive motility; ≥ 40% total motility; ≥15 × 10^6^ cells ml^−1^) as defined by established criteria (see WHO, 2010). This procedure had the approval of the Tayside Committee of Medical Research Ethics (08/S1402/6) and written consent was obtained from each donor in accordance with the Human Fertilisation and Embryology Authority (HFEA) 8th Code of Practice. Each sample was allowed to liquefy at 37°C for ∼30 min and the semen then added to a 50 ml Falcon tube containing 5 ml of HTF (see above). Since the aim was to separate motile spermatozoa from other components of the semen, this addition was undertaken gently to ensure that mixing was minimized and that the liquefied semen sample formed a distinct layer at the bottom of the tube. The tube was then inclined at 45° and incubated for 1 h at 37°C. The overlying HTF was then aspirated carefully and the motile spermatozoa that had swam into the HTF then allowed to settle into a loose pellet (1 h at room temperature). The cells were re-suspended in capacitating media and maintained at 37°C for 4 h (5% CO_2_). Capacitated cells were then re-suspended in standard bath solution and allowed to adhere to glass coverslips that were transferred to an inverted microscope where they were superfused with standard bath solution.

### Electrophysiology

The electrophysiological properties of individual spermatozoa were investigated using the whole cell recording technique (Hamill *et al.*, 1981; Kirichok *et al.*, 2006; Lishko *et al.*, 2011a). The recording pipettes (10–18 MΩ) were fabricated from borosilicate glass and normally filled with standard pipette solution. Gigaohm seals were obtained by bringing the pipette tip into gentle contact with the cytoplasmic droplet, which lies just behind the sperm head, and the patch of membrane spanning the pipette tip then ruptured by applying suction in conjunction with 1 ms voltage pulses (see Lishko *et al.*, 2010). Our standard recording conditions were designed to preserve physiologically relevant Na^+^, K^+^ and Cl^−^ gradients and *V*_m_ was held (pClamp 10 Software, Axon Instruments) at a hyperpolarized value (−92 mV) between test pulses. Initial experiments were undertaken by recording the membrane currents (*I*_m_) evoked by ramping (250 ms) *V*_m_ from −92 mV to 68 mV at 1 Hz. To analyse the results of such experiments, *I*_m_ was first normalized to input capacitance (i.e. expressed as pA pF^−1^) to ensure that variations between the sizes of different spermatozoa did not contribute to the variability in the presented data. All cited values of *V*_m_ were corrected for the liquid junction potential between the pipette/bath solutions (*E*_L_), and for the voltage drop across the access resistance (*R*_a_, 62.8 ± 0.8 MΩ, *n* = 476 cells from 29 donors). The latter correction was applied retrospectively using the expression *V*_m_ = *V*_Pip_ − *R*_a_·*I*_m_, where *V*_Pip_ is the pipette potential. Since the bath was grounded via a 4% agar/3 M KCl, bridge, the bath solution changes imposed during the present study had negligible effects upon *E*_L_. Plots showing the relationship between *I*_m_ and *V*_m_ were constructed and, unless otherwise stated, cited values of membrane conductance (*G*_m_, pS pF^−1^) are derived by regression analysis (i.e. Δ*I*_m_/Δ*V*_m_) of data recorded at positive potentials. Resting *V*_m_ was either inferred from the reversal potential (*V*_Rev_, i.e. the value of *V*_m_ at which *I*_m_ is zero, voltage clamp experiments) or measured directly by monitoring (5 KHz, data low pass filtered at 3 KHz) the zero current potential (see Hamill *et al.*, 1981). Experiments that quantified the responses to step changes in *V*_m_ were undertaken using an experimental design that employed the standard features of pClamp software (V/4 protocol) to subtract leak/capacitive currents from all recorded data. The statistical significance of differences between control/experimental values were determined tested using Student's paired (repeated measurements on the same cells) or unpaired (comparison between different groups of cells) *t*-test. The results of experiments that followed more complex protocols were analysed by one way analysis of variance (ANOVA)/Dunnet's *post hoc* test. Data are cited as mean ± s.e.m. and values of *n* refer to the number of spermatozoa in each group. All observations were confirmed using spermatozoa from at least three different donors.

## Results

### Currents evoked by voltage ramps

**Figure 1:**
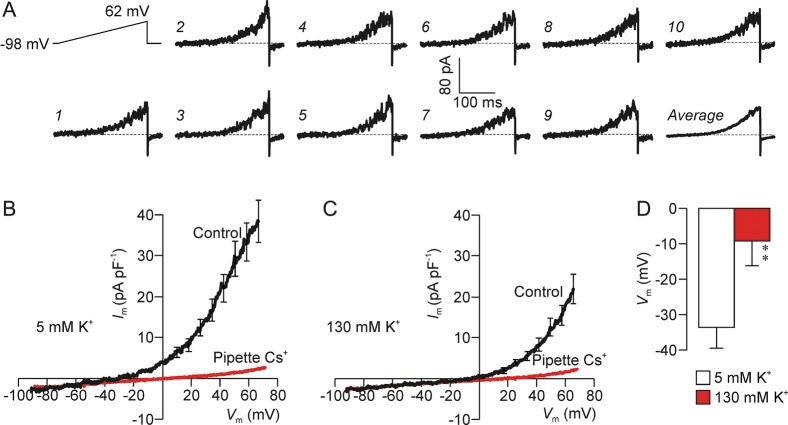
K^+^ currents in human spermatozoa. (**A**) Raw experimental traces showing the membrane currents evoked by a series of depolarizing voltage ramps (top left) that were imposed at 1 Hz. To analyse the results of such experiments, the currents evoked by successive depolarizations were pooled in order to obtain an average response for each spermatozoon (bottom right). (**B**) *I*_m_–*V*_m_ relationships quantified under standard conditions (*n* = 12) and using Cs^+^-based pipette solution (*n* = 8). (**C**) Currents recorded from the same cells after 20–30 s exposure to K^+^-rich bath solution. (**D**) Values of resting *V*_m_ estimated by regression analysis (see Materials and Methods) of data recorded using the standard pipette filling solution and during to standard (5 mM K^+^) and K^+^-rich (130 mM K^+^) bath solutions. All data shown as mean ± s.e.m.

Imposing depolarizing voltage ramps upon spermatozoa exposed to physiologically relevant Na^+^, K^+^ and Cl^−^ gradients (i.e. using standard pipette/baths solutions) consistently evoked noisy outward current. To characterize the conductance underlying this response, currents evoked by 10 successive voltage ramps were averaged (Fig. 1A) and data derived from different cells pooled and plots showing the *I*_m_–*V*_m_ relationship constructed. This analysis revealed small (1–2 pA pF^−1^) inward currents at hyperpolarized potentials whilst 25–45 pA pF^−1^ of outward current became apparent once *V*_m_ was depolarized past approximately −30 mV (Fig. 1B). Membrane conductance quantified at depolarized potentials (634 ± 85 pS pF^−1^) was 15.7 ± 2.0-fold greater than at hyperpolarized potentials (Fig. 1B; *P* < 0.001). Since seal resistance was >20 GΩ, Ohm's Law predicts that <5 pA of inward current will flow via this resistance at −100 mV, and the magnitude of the current recorded at potentials below approximately −30 mV (Fig. 1) is therefore similar to the predicted magnitude of this ‘leak current’. We therefore conclude that *I*_m_ is too small to be measured when Vm is less than −30 mV. Switching to K^+^-rich bath solution (20–30 s) depolarized resting *V*_m_ by shifting the *I*_m_–*V*_m_ relationship to the right (Fig. 1C and D) whilst replacing pipette K^+^ with Cs^+^ virtually abolished the voltage-induced outward current and depolarized resting *V*_m_ to −1.2 ± 5.8 mV (*P* < 0.002; Fig. 1B). The K^+^-rich bath solution had no effect upon the currents recorded using Cs^+^-based pipette solution (Fig. 1B and C) and this outward current must therefore be carried by K^+^. Figure 1A also shows that the recorded current consistently undershoots its basal value when *V*_m_ is repolarized after each voltage ramp. Such ‘tail currents’ (*I*_Tail_) imply the presence of voltage-gated channels that become active during the depolarization but take a finite time to close when *V*_m_ is repolarized.

### Effects of altering internal pH (pH_i_)

**Figure 2:**
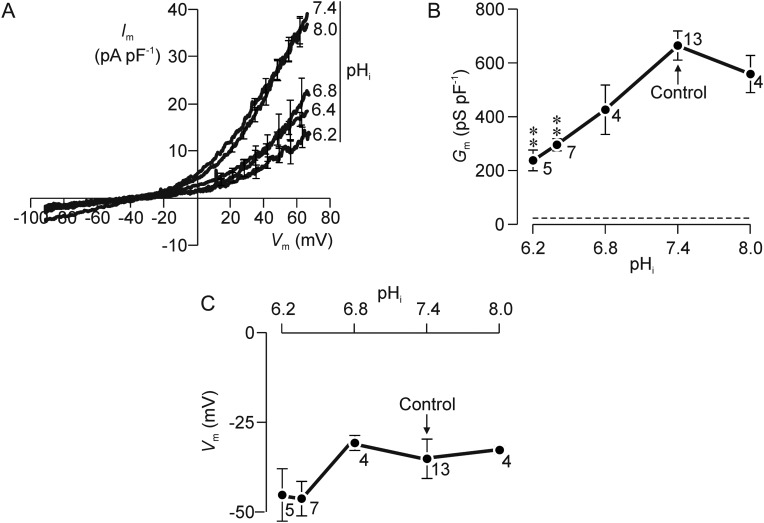
Effects of altering internal pH (pH_i_). (**A**) *I*_m_–*V*_m_ relationships quantified using pipette filling solutions that had been adjusted to a pH value ranging from 6.2 to 8.0. (**B**) The values of *G*_m_ derived by regression analysis of currents flowing at positive potentials are plotted against pH_i_; the dashed line shows the value of *G*_m_ quantified using Cs^+^-rich pipette solution. (**C**) Values of resting *V*_m_ estimated by quantifying the reversal potential are plotted against pH_i_. All data are mean ± s.e.m. and values of *n* are shown besides each point; asterisks denote data that differed significantly (*P* < 0.01, one way ANOVA/Dunnet's *post hoc* test) from the value of *G*_m_ quantified at pH_i_ 7.4.

Figure 2A shows *I*_m_–*V*_m_ relationships quantified using internal (i.e. pipette) solutions adjusted to pH values between 6.2 and 8.0. These pipette solutions were buffered with 5 mM MES/5 mM HEPES rather than 10 mM HEPES (see Materials and Methods) but the data recorded at pH_i_ 7.4 were virtually identical to the control data shown above and this modification thus has no effect upon the recorded current. These data therefore confirm that depolarization normally evokes outward current. Increasing pH_i_ to 8.0 had no effect upon the *I*_m_–*V*_m_ relationship (Fig. 2A) and thus had no effect upon *G*_m_ (Fig. 2B) or *V*_m_ (Fig. 2C). Lowering pH_i_, below 6.8 reduced *G*_m_ by ∼35% (Fig. 2B) but the residual conductance recorded under these conditions was still ∼10-fold greater than that quantified using Cs^+^-based pipette solutions (Fig. 1B). Moreover, lowering pH_i_ had no statistically significant effect upon the currents recorded at physiologically relevant potentials (i.e.–50 to 10 mV) and thus caused no change in *V*_m_ (Fig. 2*C*). The ion channels underlying the voltage-induced K^+^ current thus display only weak dependence upon pH_i_ and changes in pH_i_ therefore cause no change in *V*_m_.

### Effects of K^+^ channel blockers

**Figure 3:**
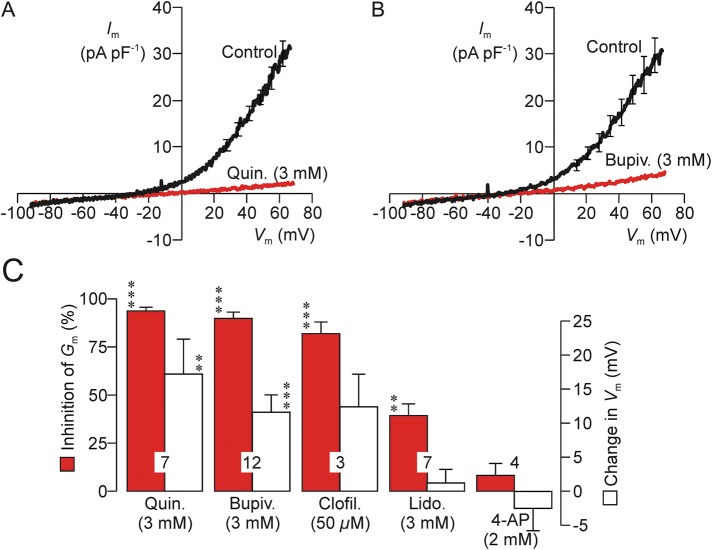
Effects of compounds that block K^+^ channels. (**A**) *I*_m_–*V*_m_ relationships quantified both under control conditions and after 20–30 s exposure to 3 mM quinidine (*n* = 7). (**B**) Results of experiments that used an identical protocol to explore the effects of 3 mM bupivacaine. (**C**) Data from experiments that explored the effects of putative K^+^ channel blockers were analysed by calculating (i) the change in *G*_m_ (% of control) induced by each test substance (filled columns), and (ii) the changes in resting *V*_m_ (i.e. the observed shift in reversal potential) induced by each test substance. Data are mean ± s.e.m. and *n* values are shown in each pair of columns. Asterisks denote statistically significant deviations from the respective control values (***P < 0.001, ** *P* < 0.01, Student's paired *t*-test).

**Figure 4:**
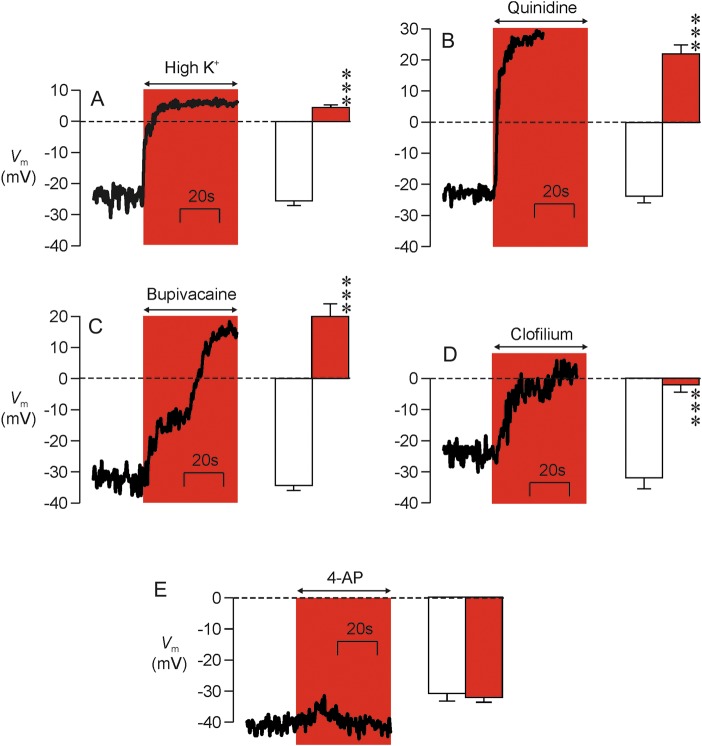
Direct measurement of *V*_m_. The left-hand part of each panel shows a continuous recording from a single cell that illustrates the effects of (**A**) K^+^-rich bath solution (*n* = 3), (**B**) 0.3 mM quinidine (*n* = 7), (**C**) 3 mM bupivacaine (*n* = 5), (**D**) 50 µM clofilium (*n* = 5) and (**E**) 2 mM 4-AP (*n* = 10) upon the zero current potential, which provides a read out of *V*_m_. The right-hand section in each panel; shows the pooled data (mean ± s.e.m) derived from the entire series of experiments. Asterisks denote statistical significant differences between the control and experimental data (*P* < 0.001, Student's paired *t*-test).

Quinidine (3 mM, Fig. 3A and C) bupivacaine (3 mM, Fig. 3B and C) and clofilium (50 µM, Fig. 3C) all caused 80–90% block of the voltage-induced outward K^+^ current, whilst 3 mM lidocaine (Fig. 3C) caused ∼30% inhibition and 2 mM 4-amino pyridine (4-AP; Fig. 3C) was ineffective (Fig. 3A–C). (Subsequent experiments showed that 0.3 mM quinidine acted as effectively as 3 mM and so this drug was used at this lower concentration in all subsequent studies.) Quinidine and bupivacaine also depolarized resting *V*_m_ (i.e. caused a rightward shift in reversal potential) and, whilst clofilium seemed to mimic this action, this effect was not statistically significant (Fig. 3C). Lidocaine and 4-AP, on the other hand, had no effect upon *V*_m_ (Fig. 3C). Examination of the control data derived from this series of experiments showed that resting *V*_m_ was normally −36.5 ± 3.3 mV and regression analysis revealed a correlation between the magnitude of the experimentally induced fall in *G*_m_ and the shift in *V*_m_ (correlation coefficient = 0.544, *n* = 33 spermatozoa, *P* < 0.001). Since these data suggest that block of the hyperpolarizing K^+^ current causes depolarization, we undertook further experiments in which resting *V*_m_ was directly monitored under zero current clamp (see Materials and Methods). These studies (i) confirmed that high external K^+^ (Fig. 4A), 0.3 mM quinidine (Fig. 4B) and 3 mM bupivacaine (Fig. 4C), but not 4-AP (Fig. 4E), depolarized *V*_m_ and, (ii) verified the depolarizing effect of clofilium (Fig. 4D). These data therefore confirm that block of the human sperm K^+^ conductance causes depolarization, but it was also clear that there were differences among the responses to the different agents tested. Clofilium thus depolarized resting *V*_m_ to ∼0 mV (Fig. 4D), whilst quinidine (Fig. 4B) and bupivacaine (Fig. 4C) shifted this potential to more positive values. Moreover, whilst the depolarizing effect of quinidine was rapid (Fig. 4B), clofilium (Fig. 4D) and bupivacaine acted relatively slowly and the response to bupivacaine was biphasic (Fig. 4C). The physiological basis of these discrepancies was not investigated further.

### Currents evoked by step depolarization

**Figure 5:**
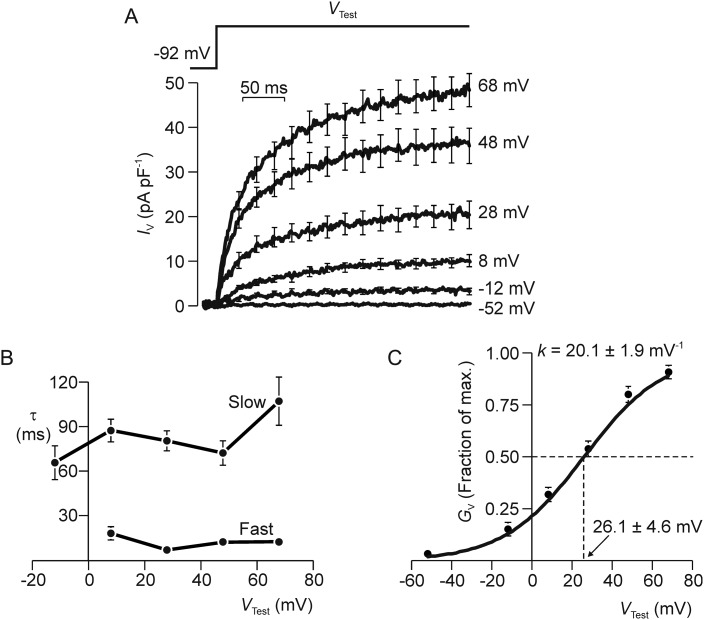
Kinetics of current activation. (**A**) Membrane currents (*n* = 7–16) evoked by maintained voltage steps to a series of test potentials (*V*_Test_). (**B**) The responses to step depolarization consistently followed time courses that were very accurately modelled as the sum of two exponential processes. The time constants (*τ*) for the fast and slow components of this response were calculated by non-linear regression and plotted against *V*_Test_. All data are mean ± s.e.m. and leak/capacitative currents were subtracted on line in order to isolate the voltage-induced component of the total membrane current (*I*_V_). (**C**) Steady-state values of *I*_V_ were quantified over the final 50 ms of each voltage step, and used to quantify the voltage-induced increase in membrane conductance (*G*_V_); the results of this analysis are plotted against *V*_Test_ and the solid line shows a solution to the Boltzmann Equation fitted to these data by non-linear regression. The Boltzmann constant (*κ*_B_) and the voltage required for half-maximal activation (*V*_50_) are presented.

To investigate the biophysical properties of the human sperm conductance further, we characterized the currents evoked by step depolarizations using an experimental protocol that allowed us to subtract the background ‘leak’ currents that flow passively through voltage-independent ion channels or across the seal resistance itself (see Materials and Methods). The important point about this experimental design is that it enabled us to isolate the voltage-induced component of the membrane current. Figure 5A thus shows voltage-evoked currents induced by stepping *V*_m_ to values between −52 and 68 mV. Depolarization consistently evoked outward current that developed over ∼300 ms (Fig. 5A) and analysis of the currents evoked by a step to 68 mV showed that the development of this current followed a time course that was accurately modelled as the sum of two exponential processes. The time constants associated with the fast (*τ*_Fast_) and slow (*τ*_Slow_) components of this response were ∼10 and ∼90 ms, respectively (Fig. 5B). Both parameters were independent of *V*_m_, and the kinetics of current activation are therefore independent of voltage (Fig. 5B). The currents evoked by depolarization to −12 mV were too small to be accurately modelled in this way and this response was best described by a single exponential with a time constant of ∼70 ms (Fig. 5B). To quantify the effect of depolarization on membrane conductance we measured the voltage-evoked currents flowing during the final 100 ms of each voltage pulse and used these data to quantify the voltage-induced increase in total membrane conductance (*G*_V_, i.e. *I*_V_/*V*_m_, Fig. 5C). Analysis of a solution to the Boltzmann Equation fitted to these data by non-linear regression showed that half-maximal activation occurred at ∼25 mV whilst the Boltzmann slope constant (*κ*_B_), which describes the channels' sensitivity to changes in voltage, was ∼20 mV^−1^ (Fig. 5C).

**Figure 6:**
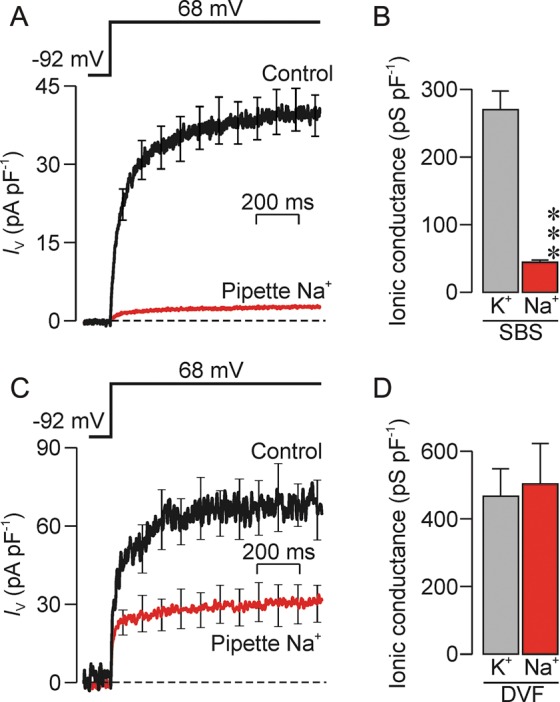
Ionic selectivity of the voltage-induced conductance. (**A**) Currents evoked by step depolarization to 68 mV were quantified both under control conditions (*n* = 18) and using Na^+^-rich pipette solution (*n* = 38) in cells exposed to the standard bath solution (SBS). (**B**) K^+^ and Na^+^ conductances quantified by analysis of data in (A). (**C**) Currents evoked using an identical voltage pulse that were subsequently recorded from the spermatozoa that were stable enough (standard pipette solution, *n* = 3; Na^+^-based pipette solution, *n* = 5) to allow the standard bath solution to be exchanged for a bath solution devoid of divalent cations (DVF). (**D**) K^+^ and Na^+^ conductances quantified by analysis of data in (C). All data are mean ± s.e.m, asterisks denote statistically significant effect of replacing pipette K^+^ with Na^+^ (***P < 0.001, Student's *t*-test).

Whilst the control data in Fig. 6A confirm that depolarization evokes outward current, this figure also includes data recorded using a pipette solution modified by replacing K^+^ with Na^+^. Whilst the response is smaller than normal, depolarization also induces outward current under these conditions. Separate experiments showed that this voltage-induced current was entirely abolished by replacing pipette K^+^ with NMDG^+^ (*n* = 9) and, as the Na^+^-, K^+^- and NMDG^+^-based pipette solutions all contained identical concentrations of Cl^−^, the fact that we observed no voltage-induced current using the NMDG^+^-based solution shows that the voltage-induced currents in Fig. 6A must be carried by cations. Moreover, since *V*_m_ was stepped to a value identical to the Na^+^ equilibrium potential (*E*_Na_, i.e. 68 mV), the control currents in Fig. 6A can only be carried K^+^, and we could thus quantify the voltage-induced increase in K^+^ conductance (*G*_K_) using the equation *G*_K_ = *I*_m_/Δ*Ψ*_K_, where *Ψ*_K_ is the electrochemical driving force on K^+^ (i.e. *V*_m_–*E*_K_). Similarly, the outward currents recorded using the Na^+^-rich pipette solution must be carried by Na^+^ since this solution was entirely devoid of K^+^. We could thus quantify the voltage-induced increase in *G*_Na_ using the equation *G*_Na_ = *I*_m_/Δ*Ψ*_Na_. Although the voltage-induced current recorded using the Na^+^ rich pipette solution was only ∼7.5% of that seen under control conditions (Fig. 6A), analysis of these data indicated that *G*_Na_ was ∼15% of *G*_K_, and the apparent discrepancy between magnitudes of the recorded currents and the calculated conductance reflects the fact that *Ψ*_Na_ is smaller than *Ψ*_K_. These experiments therefore show that *G*_K_/*G*_Na_ in depolarized cells was ∼7 (Fig. 6B). Figure 6C shows data subsequently recorded from those cells stable enough to allow the recording to be repeated 20–30 s after external Ca^2+^/Mg^2+^ had been withdrawn (see Materials and Methods). It is clear that the currents recorded using either K^+^-based and Na^+^-based pipette solutions are larger than normal and further analysis showed that *G*_Na_/*G*_K_ was now ∼1 (Fig. 6D). The modest degree of K^+^ selectivity described above therefore depends upon external Ca^2+^/Mg^2+^.

### Pharmacological profile of the outward current recorded using Na^+^-based pipette solution

**Figure 7:**
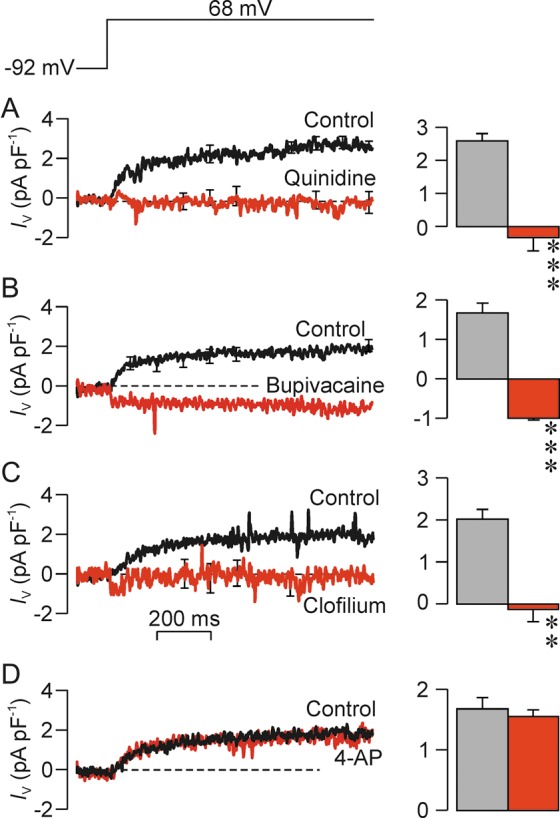
Effects of K^+^ channel blockers on the outward Na^+^ currents (*I*_Na_). Currents evoked by step depolarization to 68 mV (top panel) were recorded using the Na^+^-rich pipette solution; left-hand panels show continuous recorded of *I*_m_ whilst the right-hand panels shows mean currents quantified over the final 300 ms of the voltage pulse. In each experiment data were recorded during exposure to standard bath solution (control) and after 30–60 s exposure to 0.3 mM quinidine (A, *n* = 5); 3 mM bupivacaine (B, *n* = 6); 50 µM clofilium (C, *n* = 5) and 2 mM 4-AP (D, *n* = 5). All data are mean ± s.e.m.; asterisks denote statistically significant effects of the test substances (**P < 0.02; ***P < 0.001; Students paired *t*-test.

The data presented in Fig. 7 confirm that depolarization evoked 2–5 pA pF^−1^ of outward current when Na^+^-based pipette solutions are used, whilst analysis of data recorded after 20–30 s exposure to putative blockers showed that 0.3 mM quinidine (Fig. 7A), 3 mM bupivacaine (Fig. 7B) and 50 µM clofilium (Fig. 7C) caused >80% block of this small Na^+^ current. 4-AP was ineffective (Fig. 7D).

### CatSper blockers suppress the voltage-induced K^+^ and Na^+^ currents and depolarize resting *V*_m_

**Figure 8:**
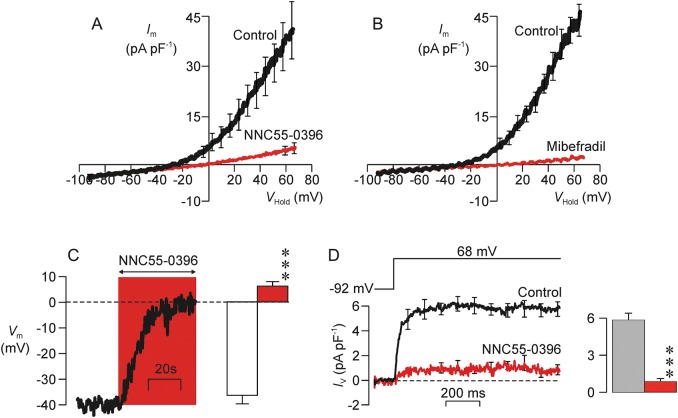
Inhibition of outward K^+^ currents by substances that block CatSper. (**A**) Relationships between *I*_m_ and *V*_m_ quantified under control conditions and after 20–30 s exposure to 2 µM NNC55-0396 (*n* = 5). (**B**) Data from experiments that used an identical protocol to explore the effects of 30 µM mibefradil (*n* = 6). (**C**) The main panels shows a continuous recording of the zero current potential and illustrates the changes in *V*_m_ that occur during exposure to 2 µM NNC55-0396 whilst the left-hand panel shows pooled data from four independent experiments. (**D**) The main panel shows mean currents evoked by a step depolarization to 68 mV recorded using the Na^+^-rich pipette solution whilst the right-hand panel shows mean currents quantified over the final 300 ms of the voltage pulse. Data were recorded during exposure to the standard bath solution (control) and after 20–30 s exposure to 2 µM NNC55-0396. All data are mean ± s.e.m and asterisks denote values that differed significantly from control (*P* < 0.001, Student's paired *t*-test).

NNC55-0396 (2 µM), a substance that blocks CatSper (Kirichok *et al.*, 2006; Lishko *et al.*, 2011a; Strünker *et al.*, 2011), caused substantial (86.6 ± 3.6%) inhibition of the voltage-induced K^+^ current (Fig. 8A) and also depolarized resting *V*_m_ from –28.2 ± 3.7 to –6.9 ± 3.8 mV (*P* < 0.005, Fig. 8A). Mibefradil (30 µM, *n* = 6), a structurally related compound that also blocks CatSper (Kirichok *et al.*, 2006; Lishko *et al.*, 2011a; Strünker *et al.*, 2011) also suppressed (94.7 ± 1.5%) the hyperpolarizing K^+^ current (*P* < 0.001) and depolarized resting *V*_m_ from –32.2 ± 2.1 to –4.2 ± 6.1 mV (Fig. 8B, *P* < 0.005). Further experiments in which *V*_m_ was monitored under zero current clamp (see Materials and Methods) confirmed the depolarizing response to 2 µM NNC55-0396 (*n* = 4; Fig. 8C). NNC55-0396 also blocked the outward Na^+^ current that is seen when Na^+^-based pipette solutions are used (Fig. 8D).

### Quinidine, bupivacaine and clofilium, but not 4-AP, block CatSper

**Figure 9:**
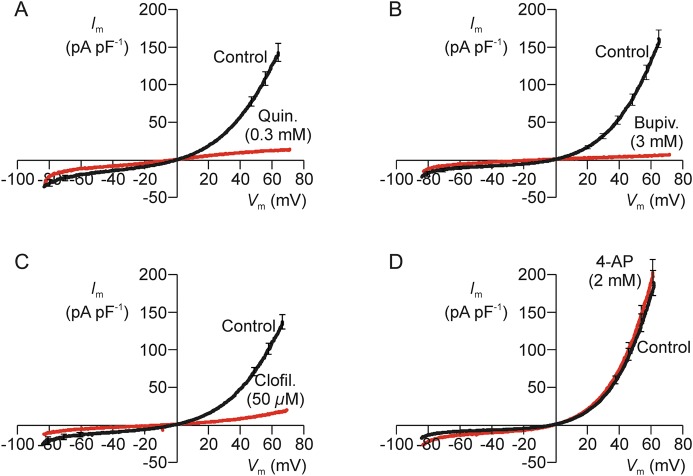
Effects of K^+^ channel blockers on the cation (Cs^+^) currents flowing via CatSper. All data were recorded using bath and pipette solutions devoid of divalent cations containing Cs^+^ as the principal cation (see Materials and Methods), and each panel shows relationships between *I*_m_ and *V*_m_ that were quantified under standard conditions (control) and after 20–30 s exposure to 0.3 mM quinidine (**A**, *n* = 8), 3 mM bupivacaine (**B**, *n* = 7); 50 µM clofilium (**C**, *n* = 5) and 2 mM 4-AP (**D**, *n* = 7).

As anticipated by earlier work (Lishko *et al.*, 2011a; Strünker *et al.*, 2011), inward and outward currents were recorded using bath and pipette solutions devoid of Ca^2+^/Mg^2+^ that contained Cs^+^ as the principal cation (see Materials and Methods), and an initial series of experiments confirmed that brief (2–3 min) exposure to 0.5 µM progesterone augmented the Cs^+^ currents flowing at negative (−86 mV; control: −56 ± 19 pA pF^−1^; progesterone: −148 ± 34 pA pF^−1^; *P* < 0.01) and positive (72 mV: control: 153 ± 33 pA pF^−1^; progesterone: 285 ± 27 pA pF^−1^; *P* < 0.001) voltages. It is now clear that the current recorded under these ionic conditions flow via CatSper (Kirichok *et al.*, 2006; Lishko *et al.*, 2011a; Strünker *et al.*, 2011), hormone-sensitive channels that become freely permeable to monovalent cations (Na^+^, K^+^, Cs^+^) if Ca^2+^/Mg^2+^ are withdrawn. The CatSper-dependent Cs^+^ current was blocked by quinidine (0.3 mM, 92.7 ± 0.7% inhibition, *n* = 8; *P* < 0.005), bupivacaine (3 mM, 98.0 ± 0.12% inhibition, *n* = 7, *P* < 0.001) and clofilium (50 µM, 87.7 ± 2.8% inhibition, *n* = 5, *P* < 0.05) whilst 4-AP had no effect (Fig. 9A–D).

### Quindine- and clofilium-induced block of the K^+^ current and CatSper

**Figure 10:**
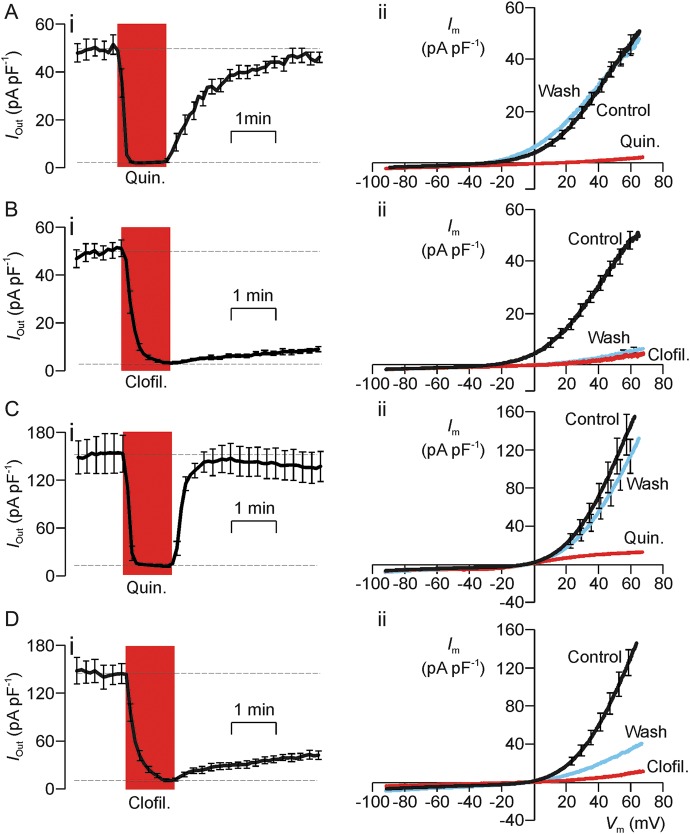
Effects of quinidine and clofilium upon the hyperpolarizing K^+^ current and the CatSper-dependent Cs^+^ current. In all experiments, membrane currents were induced by a series of voltage ramps (−92 mV to 68 mV, 5 s), and the currents flowing during the final part of each ramp then quantified as a read out of the outward current flowing at 65–68 mV (*I*_Out_). The left-hand part of each figure (i) shows the changes in *I*_Out_ induced by 1 min exposure to the test substances. The right-hand panels show *I*_m_–*V*_m_ relationships constructed using data recorded under standard conditions at the onset of the experiment (Control), once the inhibitory effect of the test substances were fully established and after the drug had been washed from the bath by 5 min superfusion with standard bath solution (Wash). (**A**) Quinidine-induced (0.3 mM) block of the hyperpolarizing K^+^ current (*n* = 5). (**B**) Clofilium-induced block of the hyperpolarizing K^+^ current (*n* = 5). (**C**) Quinidine (0.3 mM) induced block of the CatSper-dependent Cs^+^ current (*n* = 5). (**D**). Clofilium-induced block of the CatSper-dependent Cs^+^ current (*n* = 5). All data are mean ± s.e.m.

Figure 10 shows the results of experiments that compared the effects of brief (1 min) exposure to 0.3 mM quinidine and 50 µM clofilium upon the current induced by repeated ramp depolarizations. Since we have shown that the voltage-induced K^+^ current develops relatively slowly (Fig. 5), the voltage ramps used in the present studies were modified so that the cells were depolarized over 5 s. The mean current flowing during the final 200 ms of each voltage ramp was then quantified as a measure of the outward current (*I*_Out_). The magnitude of *I*_Out_ was normally ∼50 pA pF^−1^ and the data in Fig. 10A clearly show that exposure to quinidine rapidly (10–15 s) inhibits this current, but that *I*_Out_ quickly returns to its initial, control value once this drug is withdrawn. Figure 10A also includes pooled data that show *I*_m_–*V*_m_ relationships constructed using the data recorded (i) under control conditions at the onset of the experiment; (ii) once the inhibitory effect of quinidine was fully developed and (iii) 3 min after the drug was washed from the bath. Analysis of these data confirmed that quinidine causes virtually complete (95.2 ± 0.7%, *P* < 0.001, one way ANOVA/Dunnet's *post hoc* test) block of *I*_Out_ and, as anticipated, this was accompanied by depolarization of *V*_m_ (control: −19.3 ± 4.0 mV; Quinidine: −1.0 ± 0.01 mV, *P* < 0.001). Analysis of data recorded after this drug had been washed from the bath showed that *I*_Out_ had virtually returned to its control value (97.3 ± 4.1% recovery) and, similarly, *V*_m_ had returned to a value (−27.8 ± 4.4 mV) that did not differ significantly from that measured at the start of the experiment. Figure 10B shows the results of experiments that used the same method to explore the effects of 50 µM clofilium. As anticipated (see above) clofilium suppressed the recorded current although this block developed over ∼1 min and thus had a slower onset that the effects of quinidine (Fig. 10B). Analysis of data recorded once this effect was fully developed revealed substantial (91.4 ± 0.3%, *P* < 0.001) inhibition of *I*_Out_ and a clear depolarization (control: −31.9 ± 4.1 mV, clofilium: −1.0 ± 0.01 mV, *P* < 0.001). However, analysis of data recorded 5 min after this substance had been washed from the bath revealed negligible recovery of *I*_Out_ (4.0 ± 2.5% recovery) and no restoration of *V*_m_ (−1.0 ± 0.01 mV). Whilst the quinidine-induced block of the voltage-induced outward current is fully reversible, the effects of clofilium do not reverse over the time scale of the present experiments. Moreover, an initial series of experiments (*n* = 4) showed that progesterone increased the magnitude of the Cs^+^ current flowing at both positive (68–73 mV; control: 153 ± 33 pA pF^−1^; progesterone: 285 ± 33 pA pF^−1^; *P* < 0.001) and negative (−83 to −85 mV; control: −56 ± 19 pA pF^−1^; progesterone: −148 ± 34 pA pF^−1^; *P* < 0.01) potentials.

Figure 10 also includes the results of experiments that used a directly analogous protocol to explore the effects of quinidine and clofilium upon the CatSper-dependent Cs^+^ current that can be recorded under physiological conditions. These studies confirmed that 0.3 mM quinidine also causes substantial (91.0 ± 1.6%, *P* < 0.001) block of CatSper (Fig. 10C). This block had a rapid (10–15 s) onset and was almost fully (87.1 ± 9.3% recovery) reversible (Fig. 10C). Whilst clofilium (50 µM) blocked CatSper as effectively as quinidine (91.9 ± 1.2% inhibition, *P* < 0.001), full inhibition developed over ∼1 min (Fig. 10D) and, although slight recovery was seen (Fig. 10D), the currents 5 min after the drug had been washed from the bath revealed only modest (23.2 ± 4.3%) recovery. Indeed, the current recorded under these conditions did not differ significantly from the current measured in the presence of clofilium. This drug therefore causes essentially irreversible block of CatSper.

### Biophysical properties of the channels underlying the voltage-induced ‘tail current’

**Figure 11:**
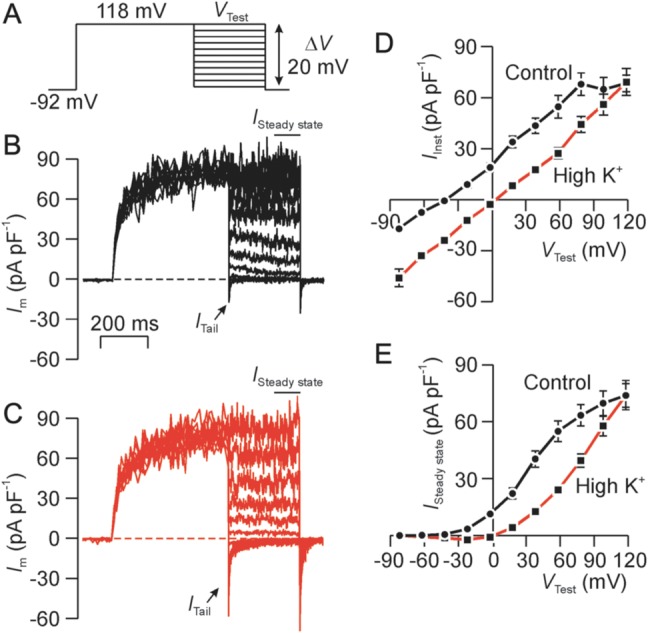
Conductive properties of the ion channels that underlie the transient tail current (*I*_Tail_). (**A**) Voltage pulse protocol used in all experiments. (**B**) Typical record showing currents recorded under standard conditions *I*_Tail_ was quantified immediately after *V*_m_ was stepped to *V*_Test_, whilst the steady-state current (*I*_Steady state_) was quantified as the mean current recorded over the final few ms of the test pulse. (**C**) Data subsequently recorded after 20–30 s exposure to K^+^-rich bath solution. (**D**) Plots showing the *I*_Tail_–*V*_m_ relationship quantified during exposure to standard bath solution (Control) and after 20–30 s exposure to K^+^-rich bath solution (High K^+^). (**E**) Plots showing the *I*_Steady state_–*V*_m_ relationship quantified during exposure to standard bath solution (Control) and after 20–30 s exposure to K^+^-rich bath solution (High K^+^). All data are mean ± s.e.m. (*n* = 5).

To explore the conductive properties of the ion channels that underlie the ‘tail’ current shown in Fig. 1A, we initially held *V*_m_ at a strongly depolarized value in order to activate the channels, and then stepped to a series of test values (*V*_Test_, Fig. 11A). Since leak/capacitive currents were subtracted (see Materials and Methods), the current measured immediately after the transition to *V*_Test_ (*I*_Tail_, Fig. 11B) reflects current flow through channels opened by depolarization. Experiments undertaken under standard conditions showed that the *I*_Tail_–*V*_m_ relationship was essentially linear (Fig. 11C) indicating that the channels do not display intrinsic rectification. Moreover, the channels cannot be K^+^ selective since *V*_Rev_ (−44.9 ± 2.9 mV) differed from *E*_K_ (*P* < 0.0001, one sample *t*-test). As we do not observe Cl^−^ current under the present conditions our subsequent analyses were based upon the assumption that these currents are carried by K^+^ and Na^+^. The channels' fractional permeability to K^+^ (*P*_K_) and Na^+^ (*P*_Na_) were therefore assigned initial, arbitrary values that were used to predict *V*_Rev_ from the Goldman–Hodgkin–Huxley (GHK) equation. The solution to this equation that best described the observed value of *V*_Rev_ was then identified by reiteratively adjusting *P*_K_ and *P*_Na_. This analysis showed that *P*_K_/*P*_Na_ was 3.0 ± 1.1. Figure 11D shows that brief (20–30 s) exposure to K^+^-rich bath solution increased the magnitude of *I*_Tail_ and depolarized *V*_Rev_ to a value close to zero (*P* < 0.001). The observed shift in *V*_Rev_ (44.1 ± 1.9 mV) was virtually identical to that predicted by the GHK equation (45 mV) for a conductance with the degree of K^+^ versus Na^+^ reported above. Further experiments (*n* = 5) used an analogous approach to measure the change in *V*_Rev_ induced by lowering bath Na^+^ to 11 mM by iso-osmotically substituting NMDG^+^, a nominally impermeant ion. This response (−15.7 ± 1.5 mV) was also virtually identical to that predicted by the GHK equation (−15 mV). The channels that underlie *I*_Tail_ thus display modest (∼3-fold) K^+^ versus Na^+^ selectivity.

Whilst repolarization consistently induced *I*_Tail_ (Fig. 11A and B), this current was transient at hyperpolarized potentials and thus decayed rapidly to a stable value that was maintained throughout the remainder of the test pulse. Analysis of the ‘steady-state’ current (*I*_Steady state_) recorded during the final few ms of each test pulse thus allows us to characterize the sustained voltage-induced outward current, and this analysis confirm that maintained depolarization induced a sustained outward current that is carried by K^+^.

### Effects of K^+^ channel blockers on the tail current

**Figure 12:**
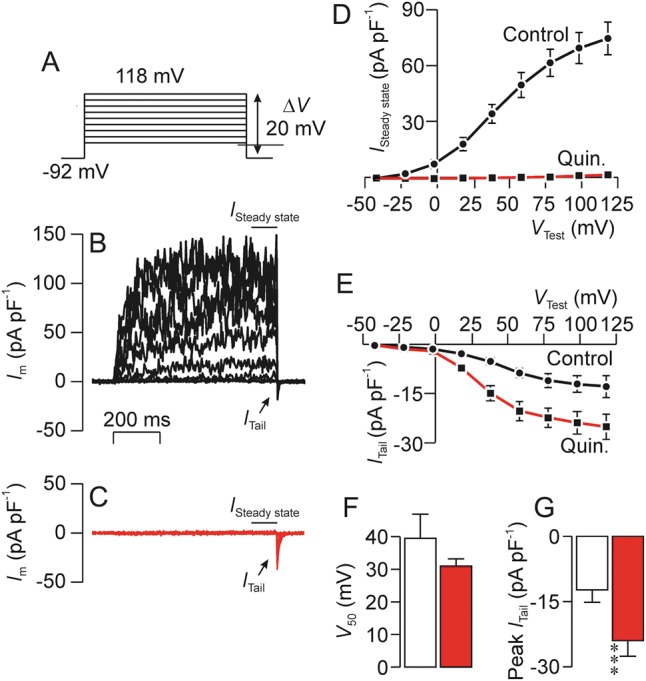
Effects of quinidine (0.3 mM) upon the sustained outward (*I*_Steady state_) and the transient tail current (*I*_Tail_). (**A**) Voltage pulse protocol used in all experiments. (**B**) Typical record showing currents recorded under standard conditions. (**C**) Currents subsequently recorded after ∼1 min exposure to 0.3 mM quinidine (Quin.). (**D**) Effects of quinidine upon the sustained outward current (*I*_Steady state_) quantified as the mean current recorded during the final few ms of each voltage pulse. (**E**) Effects of quinidine on the peak tail current (*I*_Tail_) quantified immediately after each test pulse. (**F**) Effects of quinidine upon the test voltage needed to induce half-maximal activation of tail. (**G**) Effects of quinidine upon the maximal value of *I*_Tail_. All data are mean ± s.e.m. (*n* = 10); asterisks denote statistically significant effects of quindine (***P < 0.001, Student's *t*-test).

**Figure 13:**
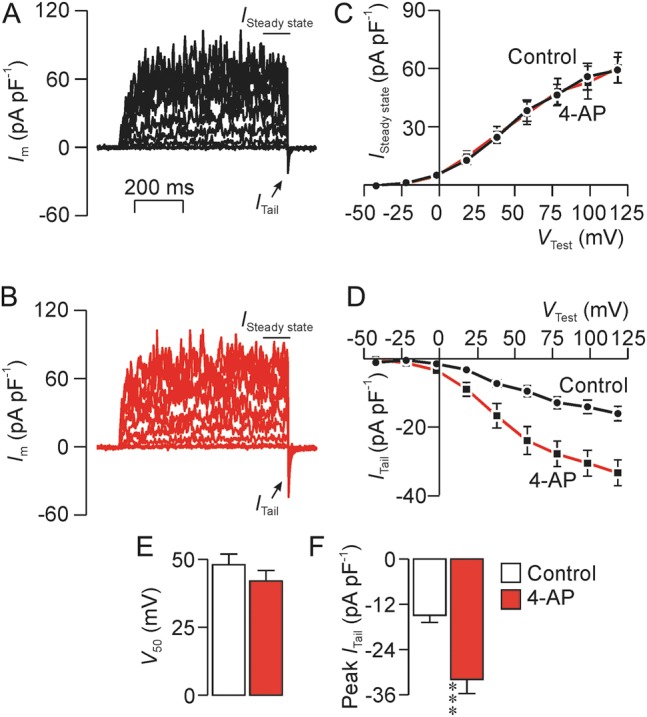
Effects of 4-AP (2 mM) upon the sustained outward current (*I*_Steady state_) and the transient tail current (*I*_Tail_). Data were recorded using a pulse protocol identical to that shown in Fig. 12. (**A**) Currents recorded under standard conditions. (**B**) Currents recorded after ∼1 min exposure to 2 mM 4-AP. (**C**) Relationships between *I*_Steady state_ and test potential (*V*_Test_) quantified under control conditions and in the presence of 4-AP. (**D**) *I*_Tail_–*V*_Test_ relationships quantified under control conditions and in the presence of 4-AP. (**E**)Effects of 4-AP upon the voltage required to induce half-maximal activation of *I*_Tail_ (*V*_50_). (**F**) Effects of 4-AP upon the maximal magnitude of *I*_Tail_. All data are mean ± s.e.m. (*n* = 8); asterisks denote statistically significant effects of 4-AP (***P < 0.001, Student's *t*-test).

The control data in Fig. 12 confirm (i) that stepping *V*_m_ to a series of test values (Fig. 12A) evokes sustained outward current (Fig. 12B) and (ii) that the subsequent repolarization induces *I*_Tail_ (Fig. 12B). Since the protocol used here (Fig. 12A) implies that *I*_Tail_ is always quantified at −92 mV the electrochemical driving forces on Na^+^ and K^+^ will be constant. The magnitude of *I*_Tail_ will therefore depend upon the extent that the channels that underlie this current had become active during the preceding depolarization. Analysis of the *I*_Tail_–*V*_Test_ relationship (Fig. 12E) therefore shows (i) that these channels normally become active at ∼0 mV, (ii) that the voltage needed for half-maximal activation (*V*_50_) is ∼40 mV and (iii) that full activation occurs at ∼75 mV (Fig. 12E). Figure 12 also includes data recorded after 20–30 s exposure to 0.3 mM quinidine (Fig. 12C) and, as anticipated (see above), this substance abolished the voltage-induced outward current (Fig. 12D). However, despite this clear and consistent effect, repolarization did induce *I*_Tail_ in quinidine-treated cells (Fig. 12C and E) and analysis of these data showed that this substance augmented this current but had no effect upon *V*_50_ (Fig. 12E–G). Figure 13 shows data from a series of experiments that used an identical protocol to explore the effects of 2 mM 4-AP. These data confirm that 4-AP does not suppress the voltage-induced outward current (Fig. 13A–C) but, despite this clear finding, 4-AP did enhance *I*_Tail_ without affecting *V*_50_. An additional series of experiments (not shown) which followed an identical approach confirmed that 50 µM clofilium abolished the voltage-induced outward current but this substance, in contrast to quinidine and 4-AP, also inhibited *I*_Tail_ (Control: *I*_Tail_ = −18.7 ± 3.7 pA pF^−1^; clofilium: *I*_Tail_ = −5.4 ± 0.5 pA pF^−1^, *n* = 5, *P* < 0.02, Student's paired *t*-test).

### Effects of progesterone on the tail current

**Figure 14:**
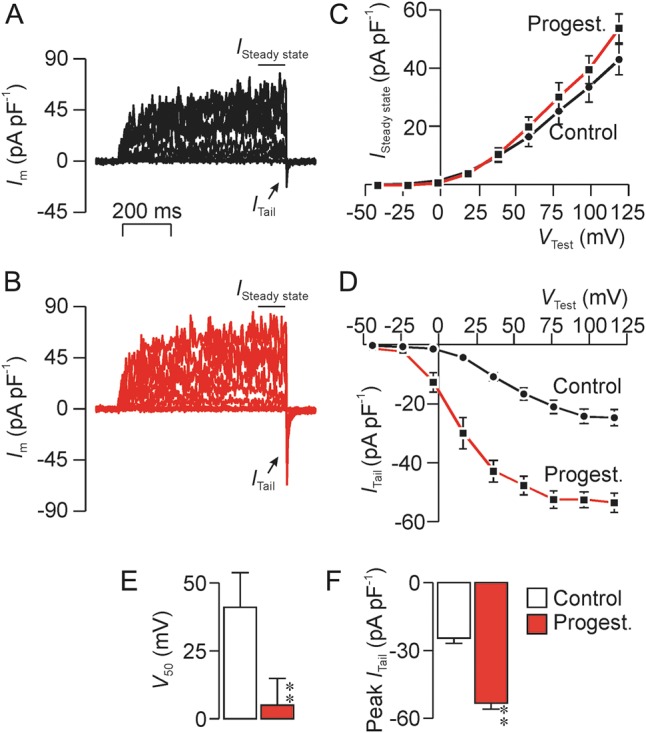
Effects of progesterone (0.5 µM) upon the sustained outward current (*I*_Steady state_) and the transient tail current (*I*_Tail_). Data were recorded using a pulse protocol identical to that shown in Fig. 12. (**A**) Currents recorded under standard conditions. (**B**) Currents recorded after ∼2 min exposure to 0.5 µM progesterone. Relationships between *I*_Steady state_ and test potential (*V*_Test_) quantified under control conditions and in the presence of progesterone. (**D**) *I*_Tail_–*V*_Test_ relationships quantified under control conditions and in the presence of progesterone. (**E**) Effects of progesterone upon the voltage required to induce half-maximal activation of *I*_Tail_ (*V*_50_). (**F**) Effects of progesterone upon the maximal magnitude of *I*_Tail_. All data are mean ± s.e.m. (*n* = 4); asterisks denote statistically significant effects of progesterone (**P < 0.02, Student's *t*-test).

Brief exposure to progesterone (0.5 µM, 2–3 min) had negligible effect upon the voltage-induced outward currents (Fig. 14A–C) but enhanced *I*_Tail_ both by augmenting the current induced by maximally effective voltage steps and by causing a leftward shift in the *I*_Tail_–*V*_Test_ relationship and so that *V*_50_ shifted from ∼40 to ∼20 mV (Fig. 14A–C).

## Discussion

The successful application of the whole cell recording technique (Hamill *et al.*, 1981) to mouse (Kirichok *et al.*, 2006; Santi *et al.*, 2009, 2010, 2013) and human (Lishko *et al.*, 2011a, b; Strünker *et al.*, 2011; Orta *et al.*, 2012; Mannowetz *et al.*, 2013) sperm has allowed great progress to be made towards identifying and characterizing the ionic currents that flow across the membranes of these cells. In most instances, these studies have used recording conditions optimized for isolation and/or enhancement of specific currents, whereas the present experiments were undertaken using intracellular and extracellular salines that preserved physiologically relevant Na^+^, K^+^ and Cl^−^ gradients. Under these conditions the dominant membrane current was a voltage-gated cation conductance with low K^+^ versus Na^+^ selectivity (∼7:1) that allowed hyperpolarizing K^+^ current to flow at potentials > ∼−30 mV. This conductance was clearly important to the maintenance of resting *V*_m_ since high external K^+^ caused depolarization. The whole cell currents described here do, however, differ slightly from those reported in another recent study of human sperm (Orta *et al.*, 2012). Although the principal aim of this study was to characterize the human sperm Cl^−^ conductance, an initial series of experiments were undertaken using a K^+^-rich pipette solution in which [Ca^2+^]_i_ was buffered to a value that approximates to the normal resting level (∼0.1 µM). However, whilst our data consistently show an outwardly rectified current which reversed at approximately −30 mV, this earlier study described an essentially linear *I*_m_–*V*_m_ relationship that reversed at approximately −12 mV with ∼200 pA of inward current at a potential of −125 mV (Orta *et al.*, 2012). However, our standard pipette solution contained only 30 mM Cl^−^ and was slightly hypotonic whilst the pipette solution used in the earlier experiments contained 130 mM Cl^−^. We chose to work under these conditions since earlier studies of epithelial cells showed that isotonic pipette solutions containing high [Cl^−^] promote cell swelling and this, in turn, can activate ‘volume-sensitive’ conductances for Cl^−^ and K^+^ (Worrell *et al.*, 1989; Macri *et al.*, 1993). Since such channels are present in human sperm (Yeung *et al.*, 2005), their activation may explain the discrepancy between the two studies. Indeed in their subsequent experiments Orta *et al.* (2012) routinely used slightly hypertonic bath solutions to prevent the activation of such conductances.

Studies of human sperm using voltage-sensitive dyes suggest that *V_m_* is normally approximately −40 mV for non-capacitated cells (Blackmore *et al.*, 1991; Linares-Hernandez *et al.*, 1998) and approximately −50 mV for capacitated cells (Patrat *et al.*, 2002) and, since similar values have been reported in mouse and bull (Zeng *et al.*, 1995; Arnoult *et al.*, 1999), capacitation seems to be associated with hyperpolarization. Although the cells used in the present study were exposed to capacitating medium, our estimates of *V*_m_ are slightly less negative than those reported in earlier studies. Since it is now clear that several key components of the capacitation process are reversible (Bedu-Addo *et al.*, 2005), it is possible that that the effects of incubation in capacitating conditions may not have been maintained during recording. Moreover, since low molecular weight substances (e.g. nucleotides, amino acids and sugars) are lost from the cytoplasm during whole cell recording (Hamill *et al.*, 1981), we cannot exclude the possibility that such substances may be needed to maintain a fully polarized membrane potential.

### Pharmacological/biophysical properties of the human sperm K^+^ conductance

Although the hyperpolarizing K^+^ current in human sperm was suppressed by acidification of pH_i_, this effect was modest and even at pH_i_ 6.2, the residual K^+^ conductance was large enough to maintain *V*_m_. In contrast, lowering pH_i_ < 7.0 depolarizes *V*_m_ of mouse sperm by inducing a profound fall in *G*_K_. The K^+^ channels in mouse are thus more sensitive to changes in pH_i_ than their human counterparts (Lishko *et al.*, 2011b). The fact that *G*_K_ displays such strict dependence upon pH_i_ in mouse implies that the cells will hyperpolarize in response to cytoplasmic alkalinization and this provides a physiological basis for at least part of the hyperpolarizing shift in *V*_m_ that occurs upon capacitation (Navarro *et al.*, 2007; Martínez-López *et al.*, 2009; Santi *et al.*, 2010; Zeng *et al.*, 2011). Whilst capacitation in human sperm also seems to involve hyperpolarization (Blackmore *et al.*, 1991; Linares-Hernandez *et al.*, 1998; Patrat *et al.*, 2002), the present data show that the mechanisms that explain this process in mouse (Navarro *et al.*, 2007; Martínez-López *et al.*, 2009; Santi *et al.*, 2010; Zeng *et al.*, 2011) cannot necessarily be applied to humans.

Our data show that the human sperm K^+^ conductance is blocked by quinidine, bupivacaine and, to a lesser extent, by lidocaine whilst 4-AP was ineffective. Moreover, experiments in which *V*_m_ was directly monitored showed that quinidine, bupivacaine and clofilium, but not 4-AP, caused depolarization, and these data clearly confirm that K^+^ channels are necessary for the maintenance of *V*_m_. These findings accord with data from mouse where the hyperpolarizing K^+^ currents display a similar pharmacological profile (Navarro *et al.*, 2007; Martínez-López *et al.*, 2009; Santi *et al.*, 2010; Zeng *et al.*, 2011). However, rather than abolishing *V*_m_, quinidine and bupivacaine shifted this potential to a positive value. It is therefore interesting that these two compounds were the most effective blockers of the hyperpolarizing K^+^ current and the fact that *V*_m_ becomes positive when *G*_K_ is blocked must indicate the presence a second conductance that mediates depolarizing current, and such current must be carried by Na^+^ and/or Ca^2+^. Whilst the present data show that *G*_K_ maintains *V*_m_ under the physiological conditions, changes to the activity of this second conductance would allow control over this potential. In this context, it is interesting that in addition to the *Slo3*-encoded K^+^ conductance, mouse sperm do appear to express epithelial Na channels (ENaC) that allow depolarizing Na^+^ currents to influence *V*_m_. Indeed, inhibition of ENaC seems to contribute to the hyperpolarizing shift in *V*_m_ that is associated with capacitation (Hernandez-Gonzalez *et al.*, 2007; Escoffier *et al.*, 2012).

Experiments in which *V*_m_ was stepped to a series of test potentials confirmed that depolarization evokes hyperpolarizing K^+^ current in human sperm. However, though human spermatozoa do express protein and mRNA encoding ‘classical’ voltage-gated K^+^ channels (Yeung and Cooper, 2001, 2008; Barfield *et al.*, 2005a, Yeung *et al.*, 2005), the currents reported here are not consistent with activity of these channels. Upon depolarization the current developed relatively slowly and the kinetics of current activation were independent of voltage. Half-maximal activation occurred at ∼25 mV whilst *κ*_B_, which describes the channel's sensitivity to changes in voltage, was ∼20 mV^−1^. Equivalent values for voltage-gated K^+^ channels are approximately −20 and ∼6 mV^−1^, respectively (reviewed by Wulff *et al.*, 2009) and, in comparison, the K^+^ channels in human spermatozoa thus display only very weak voltage-dependence. These characteristics resemble those of the K^+^ conductance found in mouse sperm (Navarro *et al.*, 2007; Martínez-López *et al.*, 2009; Santi *et al.*, 2010; Zeng *et al.*, 2011). Moreover, when pipette (cytoplasmic) K^+^ was replaced by Na^+^ depolarizing steps evoked a small outward current that displayed the same pharmacological profile as the K^+^ current. The simplest explanation of these data is that this Na^+^ current flows via the same population of ion channels as the K^+^ current. Calculation of relative permeability for K^+^ versus Na^+^ gave a value for selectivity of ∼7. Again, this resembles the characteristics of the mouse K^+^ conductance (Navarro *et al.*, 2007; Martínez-López *et al.*, 2009; Santi *et al.*, 2010; Zeng *et al.*, 2011).

### Identity of the primary K^+^ channel in mouse and human sperm

The hyperpolarizing K^+^ currents in mouse sperm is believed to flow via channels encoded by *Slo3* (*KCNMA3*) (Navarro *et al.*, 2007; Santi *et al.*, 2010; Zeng *et al.*, 2011). These channels resemble the endogenous K^+^ channels in mouse and human since (i) they are blocked by quinidine and clofilium but not by external 4-AP; (ii) are only weakly activated by depolarization and (iii) display poor K^+^/Na^+^ selectivity (Schreiber *et al.*, 1998; Martínez-López *et al.*, 2009). Moreover, like the K^+^ conductance and membrane potential of mouse sperm, *Slo3*-encoded channels are sensitive to changes in pH_i,_ an effect that reflects altered channel gating rather than an effect upon the permeability of the channel pore (Zhang *et al.*, 2006a, b). Finally, *Slo3* gene deletion abolishes the hyperpolarization seen during capacitation and mimics the effects of K^+^ channel blockade (see Barfield *et al.*, 2005a, b) by impairing progressive motility, suppressing the acrosome reaction and disrupting the control of cell volume (Santi *et al.*, 2010; Zeng *et al.*, 2011). However, despite these clear findings, heterologous expression studies show that *Slo3*-encoded K^+^ channels are virtually inactive at potentials <0 mV whilst it is abundantly clear that K^+^ currents can be recorded from mouse (Navarro *et al.*, 2007; Martínez-López *et al.*, 2009; Santi *et al.*, 2010; Zeng *et al.*, 2011) and human at such potentials (see also Lishko *et al.*, 2011b). This may reflect a requirement for interaction with the auxiliary subunit LRRC52 (leucine-rich repeat-containing protein no. 52) that is also found exclusively in male germ cells. Indeed, co-expression with of *Slo3*/*LRRC52* modifies the behaviour of *Slo3*-encoded K^+^ channels such that the current–voltage relationship more closely resembles that recorded from sperm themselves (Yang *et al.*, 2011; Yan and Aldrich, 2012).

Whilst these data are consistent with idea that the K^+^ channels in mouse and human are encoded by Slo3, recent studies have shown that charybdotoxin, paxillin and iberiotoxin all block the human sperm K^+^ conductance but have no effect upon the equivalent conductance in mouse (Mannowetz *et al.*, 2013). Since these three substances are all thought to block the channels encoded by *Slo1* and not *Slo3* (Tang *et al.*, 2010), these new data provide strong evidence that different K^+^ channel subtypes underlie *G*_K_ in mouse and human. Indeed, the fact that changes in pH_i_ had only minor effects upon the K^+^ current recorded from human sperm (see above) does tend to support this hypothesis since it is abundantly clear that the K^+^ channels in mouse sperm are very sensitive to changes in pH_i_ (Navarro *et al.*, 2007; Santi *et al.*, 2010; Zeng *et al.*, 2011). However, recent experiments that directly compared the biophysical properties of mouse and human *Slo3*/*LRRC52* showed that the human channel complex could still pass hyperpolarizing K^+^ current when pH_i_ was <7.0 (Leonetti *et al.*, 2012), a result which accords well with the K^+^ currents which we now describe in human spermatozoa themselves. The K^+^ conductance associated with mouse *Slo3*/*LRRC52*, on the other hand, was essentially inactive under such conditions (Leonetti *et al.*, 2012), a result that accords well with electrophysiological data derived from mouse spermatozoa (Navarro *et al.*, 2007; Santi *et al.*, 2010; Zeng *et al.*, 2011). The K^+^ channels encoded by human and murine *Slo3* therefore display different biophysical properties. Moreover, we also show that the human sperm K^+^ current is blocked by NNC55-0396 and mibefradil and, although these drugs are not usually considered to be K^+^ channel blockers, they do seem to block *Slo3* (Navarro *et al.*, 2007; Zeng *et al.*, 2011). Moreover, whilst *Slo1* encoded K^+^ channels display a very high degree of K^+^ selectivity (Hoshi, 2012), the hyperpolarizing K^+^ current seen during sustained depolarization flows via a population of ion channels that displayed only modest (∼7-fold) Na^+^ versus K^+^ selectivity.

### Possible involvement of CatSper

Although CatSper forms a hormone-sensitive Ca^2+^ channel under physiological conditions, this channel becomes freely permeable to monovalent cations if Ca^2+^/Mg^2+^ are withdrawn and its activity has thus been assessed by monitoring Cs^+^ current that can flow through the channel under divalent-free conditions (Kirichok *et al.*, 2006; Lishko *et al.*, 2011a). This characteristic of CatSper, which is a result of divalent cation binding within the channel pore, can explain earlier observations which showed that divalent cation-free (or depleted) medium causes enhanced Na^+^ influx and depolarization of human sperm (Gonzáles-Martinez, 2003; Torres-Flores *et al.*, 2011) and can also account for the loss of K^+^ versus Na^+^ selectivity that we observed in medium devoid of divalent cations medium. However, NNC55-0396 and mibefradil, structurally related compounds that block CatSper, suppressed the hyperpolarizing K^+^ recorded under standard (physiological) conditions. Moreover, the effects of quinidine, bupivacaine, clofilium and 4-AP upon the CatSper-dependent Cs^+^ current seen under divalent-free conditions were indistinguishable from their effects on the K^+^ current recorded under standard conditions. It is therefore interesting that recordings of currents from sperm of mice null for *Slo3* and/or *CatSper1* show that hyperpolarizing K^+^ current can flow though CatSper at potentials greater than ∼30 mV (Zeng *et al.*, 2011, 2013). Moreover, although mouse KSper and CatSper thus appear to share many pharmacological features, the clofilium-induced block of Slo3 was essentially irreversible whilst this drug's effect on CatSper reversed rapidly (Navarro *et al.*, 2007; Zeng *et al.*, 2011). Since this seems to provide a way of distinguishing between the two channel types (Zeng *et al.*, 2011), we undertook a detailed series of experiments that compared the effects of quinidine and clofilium upon the hyperpolarizing K^+^ current and the CatSper-dependent Cs^+^ currents in human sperm. Quinidine caused reversible block of both currents, consistent with data from mouse (Navarro *et al.*, 2007; Zeng *et al.*, 2011) but unlike the mouse, clofilium caused essentially irreversible block of both currents. Thus, in human sperm it is very difficult to distinguish K^+^-channel currents from monovalent CatSper on pharmacological grounds and, as in mouse (Zeng *et al.*, 2011, 2013), a part of the hyperpolarizing K^+^ current may flow via CatSper. However, not all of our data were consistent with this hypothesis since progesterone augmented the CatSper-dependent Cs^+^ current (Kirichok *et al.*, 2006; Lishko *et al.*, 2011a; Smith *et al.*, 2013) but had only a negligible effect upon the hyperpolarizing K^+^ current. This result therefore suggests that most of the K^+^ current must flows via a separate population of K^+^ channels.

### The tail current

Repolarization of *V*_m_ after a test depolarization consistently induced transient inward current (*I*_Tail_), and ionic substitution studies showed that these currents flowed via channels that were less K^+^ selective than those underlying the sustained outward current. Furthermore, quinidine augmented *I*_Tail_ despite causing full block of the hyperpolarizing K^+^ current whilst 4-AP also augmented *I*_Tail_ with no effect upon the sustained K^+^ current. Clofilium, on the other hand, blocked both currents. There are therefore clear pharmacological and biophysical differences between the channel populations that underlie these two currents and depolarization must therefore activate at least two K^+^-permeable channel types. As far as we are aware, this is the first evidence that quinidine and 4-AP can activate any type of ion channel and these unusual responses could be highly significant since both substances can induce a ‘hyperactive’ pattern of motility (Barfield *et al.*, 2005a; Alasmari *et al.*, 2013b). Earlier studies have assumed that quinidine caused hyperactivation by blocking K^+^ channels (Barfield *et al.*, 2005a) whilst the effects of 4-AP have been attributed to changes in pH_i_ and the mobilization of Ca^2+^ from an intracellular store (Alasmari *et al.*, 2013b). The fact that these substances can both activate ion channels raises the possibility that the channel underlying *I*_Tail_ may contribute to the control of motility. Moreover, progesterone, which had no effect upon the sustained outward K^+^ current but did augment *I*_Tail_, induces hyperactivation in a proportion of human sperm (Uhler *et al.*, 1993; Fabbri *et al.*, 1998; Teves *et al.*, 2006; Sagare-Patil *et al.*, 2012; Alasmari *et al.*, 2013a, b). This response to progesterone may be critical for progress through the female tract and successful interaction with the egg and it is therefore interesting that spermatozoa from men with clinically identified fertility defects show that impaired activation by progesterone and 4-AP correlate well with reduced fertilization capacity (Alasmari *et al.*, 2013a).

The ion channels that underlie *I*_Tail_ displayed weak dependence upon *V*_m_ and, since these channels are normally inactive at approximately −30 mV, current flow through these channels cannot contribute to the resting membrane potential under the conditions of the present experiments. However, it is possible that the activity of these channels may be modified by diffusible factors that would be lost from the cytoplasm once the whole cell recording configuration is established (Hamill *et al.*, 1981) and we therefore cannot exclude the possibility that these channels may be important to the control of *V*_m_ in intact spermatozoa. However, it is interesting that, as well as increasing the magnitude of *I*_Tail_, progesterone caused a hyperpolarizing shift in *V*_50_ that allowed the *I*_Tail_ to be activated by weaker depolarizations. Progesterone-induced activation of CatSper is now well documented and this response has been studied by quantifying changes to the Cs^+^ current recorded under divalent-free conditions or to the current carried by Ba^2+^ (Lishko *et al.*, 2011a; Strünker *et al.*, 2011). We believe that our data are the first to show a progesterone-induced change to the conductive properties of spermatozoa exposed to quasi-physiological ionic gradients. Whilst the biological significance of this novel response is presently unknown, the importance of progesterone to the control of motility makes it important to characterize the progesterone-sensitive channels more fully and to establish the extent to which other substances that control sperm motility can influence their activity.

## Summary

**Figure 15:**
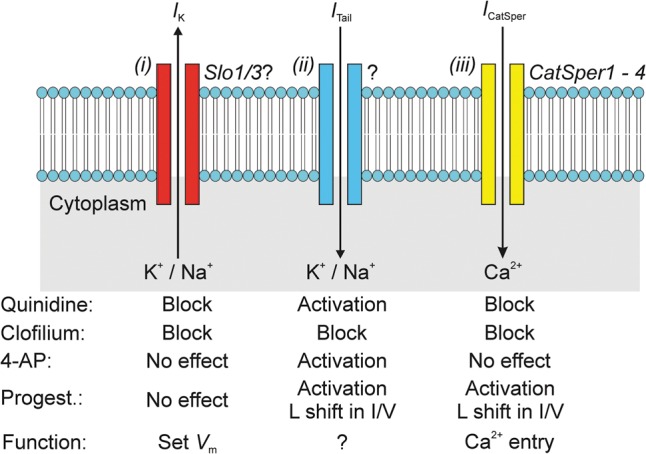
Overview of cation-permeable channels in the membranes of human spermatozoa. Electrophysiological studies of human sperm have now identified (i) a poorly selective K^+^ conductance that appears to set the resting membrane potential by allowing hyperpolarizing K^+^ current (IK) to flow across the membranes of cells depolarized past approximately −30 mV. (ii) An unidentified channel that underlies the transient inward ‘tail’ current (*I*_Tail_) that is seen upon repolarization, although the function of this conductance is unknown, the fact that it is activated by progesterone raises the possibility that it may form part of the mechanism that allows spermatozoa to respond to this female hormone. (iii) The spermatozoon cation channel (CatSper) appears to be Ca^2+^-selective under physiological conditions, although it is freely permeable to Na^+^, K^+^ and Cs^+^ in the absence of divalent cations. The effects of a range of pharmacological agents are summarized in the lower part of the figure. Each identified conductance has a characteristic pharmacological signature indicating that they must be associated with different ion channels.

Electrophysiological studies of mouse sperm (Kirichok *et al.*, 2006; Navarro *et al.*, 2007; Santi *et al.*, 2010; Zeng *et al.*, 2011) have led to the identification of two cation-permeable conductances. The first of these is a pH-sensitive K^+^ conductance that sets the membrane potential and is almost certainly encoded by *Slo3*/*LRRC52*, whilst the second is a Ca^2+^-permeable channel encoded by members of the CatSper gene family (Ren *et al.*, 2001). Although other cation-permeable conductances have been identified (see, for example, Felix *et al.*, 2002; Acevedo *et al.*, 2006; Martínez-López *et al.*, 2009), studies of knockout mouse indicate that it is *Slo3*/*CatSper* that dominate the conductive properties of murine sperm (Zeng *et al.*, 2013). The present electrophysiological studies of human sperm exposed to ‘physiological’ ionic conditions have identified a K^+^ channel that is weakly activated by voltage (Fig. 15) and this conductance is broadly similar to that recently documented in separate studies (Mannowetz *et al.*, 2013). However, whilst it has been suggested that this may flow via channels encoded by *Slo1* (Mannowetz *et al.*, 2013), the poor ionic selectivity and unusual pharmacological profile which we report are not consistent with this hypothesis. Moreover, we also show that depolarization activate a second voltage-dependent conductance that displays very poor K^+^ selectivity and is subject to rapid inactivation. This current has a different pharmacological profile to both the sustained outward K^+^ current and the CatSper-dependent Cs^+^ current but, like CatSper, shows both stimulation and leftward—shift of *I*–*V* relationship in the presence of progesterone (Fig. 13). This previously undocumented conductance may thus play an important role in mediating the physiological effects of this hormone.

## Authors' roles

S.A.M. performed all of the patch clamping and sperm function experiments, the initial analysis of the data and was critically involved in the experimental design. S.M.W., S.J.P. and C.L.R.B. were involved in the design of the study and obtained funding for the experiments. The initial funding was supported by grants from NHS Tayside, Infertility Research Trust (Barratt PI) and the Wellcome Trust (Publicover and Barratt PI). Additional funding was provided by MRC (MR/K013343/1, Wilson PI). S.M.W. and S.A.M. performed the detailed data analysis of the electrophysiological data. All authors contributed to the construction, writing and editing of the manuscript. All authors approved the final manuscript for submission.

## Funding

This study was made possible by grants from the Wellcome Trust (086470), the Infertility Research Trust, NHS Tayside and the Medical Research Council (MR/K013343/1). Funding to pay the Open Access publication charges for this article was provided by Durham University.

## Conflict of interest

None declared
